# Implementing Large Language Models to Support Misconception-Based Collaborative Learning in Health Care Education

**DOI:** 10.2196/81875

**Published:** 2026-01-16

**Authors:** Brandon C J Cheah, Shefaly Shorey, Jun Hong Ch'ng, Chee Wah Tan

**Affiliations:** 1 Department of Microbiology and Immunology, Yong Loo Lin School of Medicine, National University of Singapore, 5 Science Drive 2, Singapore, 117545, Singapore, 65 81503939; 2 Department of Pharmacy and Pharmaceutical Sciences, National University of Singapore, Singapore, Singapore; 3 Alice Lee Centre of Nursing Studies, Yong Loo Lin School of Medicine, National University of Singapore, Singapore, Singapore

**Keywords:** large language model, refutation text, collaborative learning, health care education, misconception-based learning, LLM

## Abstract

This paper proposes a framework for leveraging large language models (LLMs) to generate misconceptions as a tool for collaborative learning in health care education. While misconceptions—particularly those generated by AI—are often viewed as detrimental to learning, we present an alternative perspective: that LLM-generated misconceptions, when addressed through structured peer discussion, can promote conceptual change and critical thinking. The paper outlines use cases across health care disciplines, including both clinical and basic science contexts, and a practical 10-step guidance for educators to implement the framework. It also highlights the need for medium- to long-term research to evaluate the impact of LLM-supported learning on student outcomes. This framework may support health care educators globally in integrating emerging AI technologies into their teaching, regardless of the disciplinary focus.

## Introduction

In an age where the availability of information is the norm and not the exception, misinformation from a range of information sources—including social media—can dominate the information landscape. Potential misinformation exists in the medical world, given the increasing rates of knowledge dissemination and the presence of potentially misleading information sources.

Medical practitioners and health professionals are not exempt from its effects, with the consequences having wide-reaching implications. An example of a high-profile case where physicians generated harm due to misinformation includes the physician-authored Great Barrington Declaration during the COVID-19 pandemic, which may have potentially increased resistance to lockdown policies in the United States and Europe [1]. Other examples include a Houston-based physician tweeting in 2022 to her account of more than 50,000 followers that there were no medically valid reasons for vaccination at the time [2], or physician-promoted anti-diet movements supporting the consumption of discretionary foods [3]. Medical training must therefore explicitly cultivate the capacity to detect and dismantle misinformation.

To address misconceptions stemming from misinformation, examples of current approaches by medical educators include manual assessment of student answers in examinations [4] and in-class polling [5] to identify misconceptions for use in misconception-based learning. However, these methods assume that (1) various student cohorts over time share similar misconceptions, without potential idiosyncratic variability in thought patterns; (2) educators have a breadth of experience with multiple cohorts; and (3) educators have the time capacity to generate multiple misconceptions in-depth. These assumptions may not hold true in practice, ultimately limiting the scalability of traditional misconception-based learning to educator experience.

The advent of ChatGPT and other large language models (LLMs) fueled by artificial intelligence (AI) presents opportunities to augment misconception-based learning. LLMs have demonstrated potential to personalize learning experiences, offer instant feedback and enhance student engagement [6]. Within misconception-based learning, LLMs offer the potential to both assist educators in generating misconceptions and students in identifying inaccurate thought patterns through feedback. However, limited research exists on LLM-assisted misconception-based learning in health care education. Therefore, key questions arise: (1) how do LLMs fit within the process of misconception-based learning; and (2) how can LLMs be applied in the context of misconception-based learning in health care education? To address the research gap, this paper serves as a theoretical foundation and a conceptual framework with potential cases to apply LLMs in health care education.

## Theoretical Premise of Misconception-Based Learning

Misinformation, and later entrenched misconceptions, can result from cognitive biases [7] and the lack of critical thinking [8]. It is important to note that scholarly debate exists on the use of the word misconception, particularly surrounding its definition and appropriateness of use [9]. Here misconceptions are referred to in the lens of the Piagetian constructivist theory, which sees misconceptions as a part of active constructions of students’ mental models [10]. Misconceptions, in this context, are not deficiencies in knowledge but rather a gap between the student’s perceptions and scientifically accurate information. To be more effective, educators could elucidate and challenge misconceptions early in the process of knowledge acquisition.

One of the foundations of health care professions education lies in conceptual acquisition. Conceptual acquisition could range from logic-derived processes (ie, mechanics of inspiration and breathing in physiology) to memory-based internalization (ie, eukaryotic transcription factors in molecular biology). Misconceptions would thus be the students’ prior beliefs and constructed mental models that contradict accepted scientific concepts [11]. In health care professions, not addressing misconceptions originating from education may have ramifications on clinical reasoning and patient safety [12].

**Figure 1.**
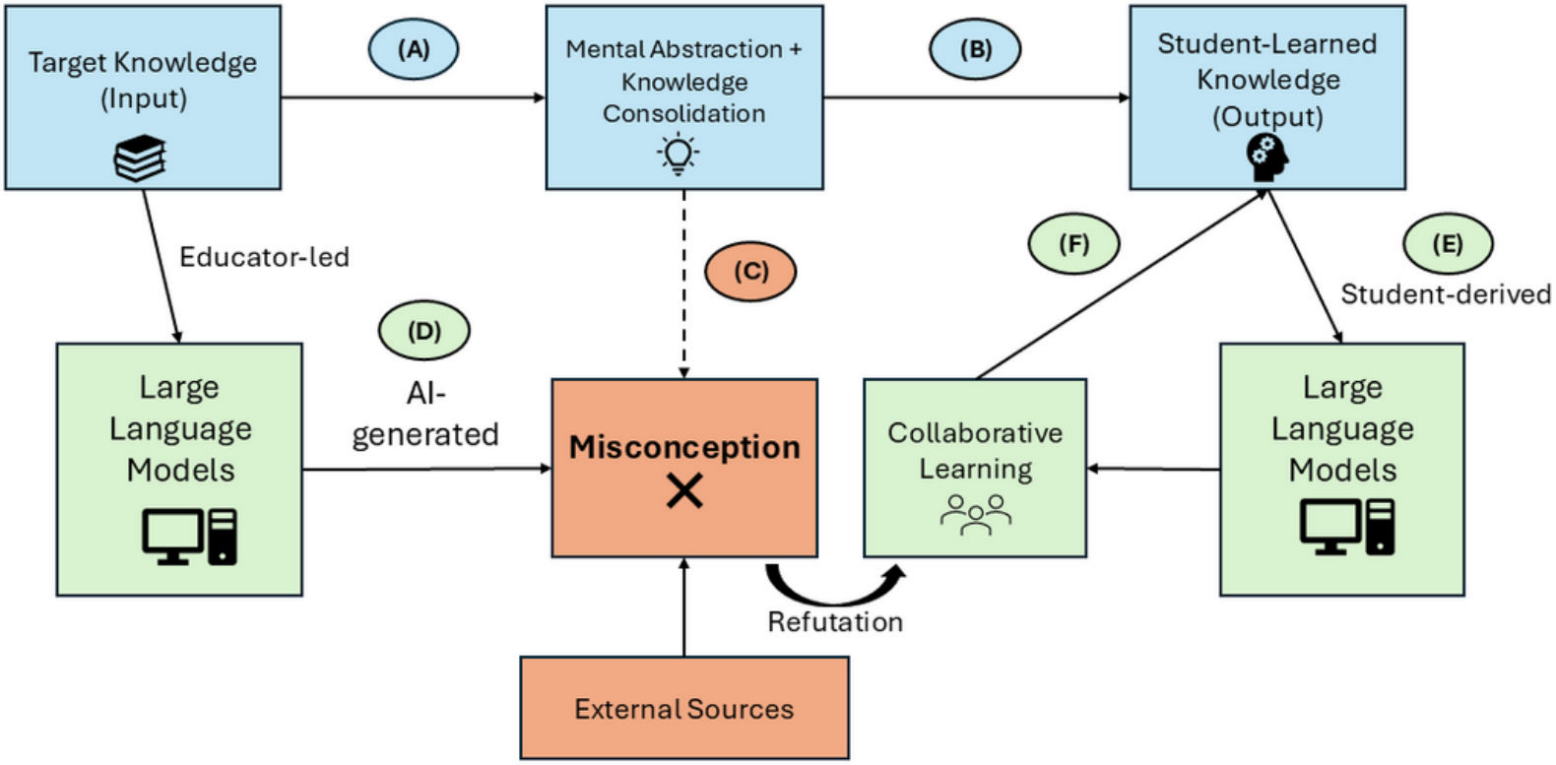
A framework for LLM-augmented misconception-based collaborative learning. LLM: large language model.

Figure 1 presents a simplified model of the process surrounding scientific conceptual acquisition. The assumed educational goal is the possession of student-learned knowledge supported by factual accuracy (output). The student is first given an input of target knowledge either through didactic or autodidactic methods. Given that the target knowledge input in health sciences can be highly conceptual, which has little to no attribution to sensorimotor information [13], the student is likely to begin the process of mental abstraction that includes simplification and consolidation of knowledge (labeled (A) in Figure 1). Conceptual acquisition could occur in a mechanistic process, with the assembly of smaller and cognitively plausible concepts as a bootstrap for developing larger concepts [14].

Ideally, the iterative process of conceptual acquisition is factually accurate, with the student utilizing prior learned knowledge to acquire new concepts, a core component of critical thinking (labeled (B) in Figure 1). However, a student is likely to develop misconceptions due to a continuously evolving, plausible intuitive interpretation of the concept according to lived experiences (denoted by the dotted arrow labeled (C) in Figure 1) [15]. Misconceptions can interfere or inhibit with the further process of mental abstraction, given that the student is likely to form a false belief that the concept learned is accurate, resulting in a persistent misconception.

One of the ways to manage misconceptions arising from the process of scientific conceptual acquisition is to pre-emptively expose students to misconceptions in the classroom and systematically debunk such misconceptions. A method to which misconception-based learning can be applied in practice is using refutation text. Refutation text is a misconception-centric pedagogical method first rooted in theories of conceptual accommodation in science [16]. Conceptual accommodation is the internalization of new concepts upon facing limitations in utilizing their existing conceptions to solve problems [17]. Conceptual accommodation, therefore, is the goal of refutation text and misconception-based learning.

Three components work together to form the refutation text pedagogical method: (1) stating common but inaccurate knowledge held by the student; (2) explicit indication of the incorrect aspects of said knowledge; and (3) stating factually correct information with supporting explanation and applications [18]. The advantages of refutation text over traditional text or didactic instruction include explicit mention of assumed prior incorrect beliefs, and increased propensity for conceptual acquisition even for students with low prior topic knowledge [19]. Given that students may find it challenging to state their own misconceptions held, the educator would be the primary source of providing the refutation text. Misconception exposure and the scientific method have already been in practice within the National University of Singapore common curriculum [20].

## Integrating LLMs: An LLM-Augmented Misconception-Based Collaborative Learning Approach

There are two potential areas for LLM augmentation: educator-led or student-derived inputs of misconceptions into LLMs (denoted by the parts labeled (D) and (E), respectively, in Figure 1). In the educator-led case, LLMs can be used to simulate common student misconceptions. Educators can input a defined set of assessment-relevant concepts and prompt the model to generate plausible errors, providing a basis for targeted instruction. Techniques in prompt engineering include few-shot prompting [21], which is the provision of additional examples of desired tasks by the user in follow-up prompts. Given that LLMs predict appropriate outputs based on patterns in training data, providing structured follow-up prompts such as through few-shot prompting can help refine the scope and relevance of these generated misconceptions. For more complex cognitive processes, such as clinical reasoning, advanced strategies like chain-of-thought prompting—which simulates step-by-step heuristic thinking—can be employed to trace and identify potential misconceptions within the reasoning pathways of clinicians-in-training [21].

In the student-derived case, LLMs can drive deeper engagement between students and the course material. Prior student examination answers, or reference texts for the concepts, could be supplied to an LLM along with a pre-defined prompt to role-play as a junior trainee. The students could then begin a Socratic-style conversation with the LLM, identifying misconceptions based on the LLM’s questions. Afterwards, the students could collaboratively correct the misconception and explain the correct understanding to the LLM. Such dialogic engagement could encourage metacognitive awareness, sustain curiosity due to the personalized nature of responses, and foster deeper conceptual understanding.

LLM-generated misconceptions complement student misconceptions revealed through assessment responses. Importantly, however, LLM-generated misconceptions may not be universally applicable across all student populations or educational contexts. As such, the lecturer’s role includes adapting LLM-generated content to suit specific curricular needs, positioning the LLM as a quasi-teaching assistant in the generation of discussion prompts.

Overall, this framework in Figure 1 outlines the approach of LLM-augmented, misconception-based collaborative learning (CL). In addition to saving educator time, reducing reliance on educator experience, and identifying idiosyncratic student misconceptions, LLMs can also drive deeper engagement between students and the learning material. Educators could then shift their focus toward fostering higher order thinking skills aligned with the upper levels of Bloom’s Taxonomy, though the extent of this shift will depend on the course’s learning objectives and institutional priorities. Additionally, student-led presentation on evaluating misconceptions generated by AI can integrate into existing pedagogical approaches on the flipped classroom, encouraging student autonomy. Ultimately, the flexibility in this framework’s extent of implementation potentially allows for a variety of use cases.

## The Utility of CL in LLM-Assisted Misconception-Based Learning

Refuting misconceptions generated by LLMs could be achieved in CL settings, such as tutorials or workshops. CL emphasizes consensus building and collective problem-solving within a peer group, in contrast to individualistic or competitive approaches. By shifting learners from passive to active modes of engagement, CL promotes critical thinking, deeper conceptual understanding, and the development of social support networks [22].

In the context of addressing misconceptions, CL provides a structured environment for students to identify, articulate, and refute conceptual errors. A three-step approach can be used to scaffold this process: (1) students first work individually to pinpoint and justify any conceptual flaws within a statement; (2) small groups are then allocated time to discuss and contrast these ideas with factually accurate knowledge, refining their reasoning through peer interaction; and (3) groups present their consensus and rationale to the larger class, facilitating the synthesis and reinforcement of accurate understanding. After the group presentation, educators could identify strengths and weaknesses in the student groups’ arguments. Additionally, LLMs could provide supplemental feedback alongside educators’ comments on the group’s quality of arguments. CL can thus have positive feedback on student-learned knowledge and conceptual retention (denoted by the part labeled (F) in Figure 1).

CL can present a risk of unmoderated reinforcement of misconceptions in LLM-supported environments through distractor information. Distractor information generated by LLMs adds unnecessary extraneous cognitive load to a student’s working memory. This generates a quasi-redundancy effect that involves processing unnecessary or misleading information, possibly reducing learning efficiency [23].

However, the presence of CL here can be a crucial first line “guardrail” to the accuracy of knowledge. Through peer discussion, learners can identify and correct hallucination errors, making CL an essential rather than peripheral component of the learning process. CL has shown to be resource efficient in digitally enabled classroom settings when addressing misconceptions [24]. Educators could then review the understanding of the student group, acting as a second “guardrail” to address problematic misconceptions. Educators could further explore initiatives to scaffold understanding through diagnosis of student understanding and reinforcing understanding within CL groups with learning checkpoints [25].

## Operational Considerations and Obstacles for Implementation

A key challenge in applying LLMs to misconception-based CL lies in data quality. LLMs learn from data patterns, and their performance depends critically on the quality and reliability of training materials [26]. Low-quality or inappropriate data can result in hallucinations or the generation of misconceptions that misrepresent core concepts. To mitigate this, domain-specific LLM architectures could be trained to draw exclusively from vetted, purpose-built educational resources such as notes, textbooks, or slides. A cost-efficient alternative for developing domain-specific LLMs is to input relevant educational resources into general-purpose models, such as GPT-4o, prior to prompt generation—though this approach may yield less domain-specific or contextually relevant outputs.

Providing high-quality data for LLM training is especially vital in health care education, where complex competencies such as clinical reasoning and clinical history taking have significant patient safety implications. In these contexts, custom LLM pipelines that utilize high-quality data—including pre-existing manual diagnostic frameworks currently in use by clinicians—are required. However, given present concerns on data security and ethical use, such data must be rigorously vetted for content, suitability, and compliance with privacy regulations before it is used to support learning and assessment. Privacy breaches in AI-assisted learning environments could compromise patient confidentiality and institutional trust in patient data security. Any adoption of LLMs in medical curricula must therefore follow the same rigor as clinical data governance.

Additionally, given that there is an absence of gold standards for clinical reasoning pipelines, it may be challenging in practice to develop an AI model specifically for clinical reasoning. Such pipelines of reasoning could be obtained from experienced clinicians for database construction, which would lead to greater quality of input data for LLMs to generate misconceptions for use in education.

While LLMs can generate customized feedback, pitfalls of LLMs in education include its potential to mislead students with false answers under the pretense of conversational and easy-to-understand responses [27]. However, a learning opportunity exists amidst the discourse, that is, how might we, as educators (1) utilize such misconceptions as a teaching opportunity, and (2) demonstrate the value of critical thinking and source evaluation in the process? A shift in the paradigm of conceptual acquisition can be achieved by seeing LLMs not as a source of harm, but as a pedagogical opportunity. This is pertinent in the health care education context, as hallucinated misinformation generated by LLMs may reinforce clinically unsafe assumptions if students are not trained to critically verify references.

Recent advances in ChatGPT-4o functionality include a deep research mode, which attempts to provide source references for the content it generates. However, because many publications remain behind paywalls and inaccessible to LLMs, the tool may still produce inaccurate citations or “hallucinated” references despite this feature. This limitation presents an opportunity for educators to help students critically evaluate the credibility of retrieved materials, distinguishing peer-reviewed from non-peer-reviewed sources and identifying instances of fabricated references.

## Use Cases for LLM-Assisted Misconception Generation

Educators seeking to use LLMs such as ChatGPT for generating misconceptions can employ role-play within a single prompt, followed by targeted clarification prompts to leverage the few-shot learning capabilities of LLMs. Table 1 illustrates example use cases across disciplines including pharmacy, nursing, medicine, molecular biology, and microbiology, highlighting the potential application of this approach to both the practice-oriented and basic science domains of health care education. Table 1 only displays the initial prompt and excludes any subsequent prompts for elaboration based on the individual educator’s expertise and use needs.

**Table 1.** Example use cases generated by GPT-4o for large language model (LLM)–assisted misconception-based collaborative learning.

Discipline	Concept(s)	Prompt	Learning goal	ChatGPT-4o output
Pharmacy	Hepatic extraction ratio	I am an educator within the pharmacy department of my university. I would like to teach the concept of hepatic extraction ratio. Come up with likely misconceptions that students may possess.	Differentiate between pharmacokinetic parameters of hepatic extraction ratio and hepatic clearance.	Misconception about hepatic extraction ratio: “The hepatic extraction ratio is the same as hepatic clearance.” Why it’s wrong: Hepatic clearance depends on both hepatic blood flow and extraction ratio, while the hepatic extraction ratio (HER) is only the fraction of drug removed from the blood during a single pass through the liver. So, clearance can vary even if HER is constant, and vice versa.
Nursing	High-flow oxygen therapy	I am a nurse educator in my university’s school of medicine. I am trying to convey that not all patients benefit from high-flow oxygen therapy, and that post-operation patients should be given fluid intake. Come up with likely misconceptions that students may have about this concept.	Apply clinical reasoning to determine when high-flow oxygen is indicated or contraindicated. Explain the effects of the lack of fluid intake post-surgery.	Common misconception about high-flow oxygen therapy: “All patients with low SpO_2_^a^ need high-flow oxygen.” Why it’s wrong: Not all hypoxic patients benefit from aggressive oxygen therapy (eg, patients with COPD^b^ at risk for hypercapnia). Reinforce understanding by highlighting clinical scenarios where high-flow oxygen is beneficial (eg, severe hypoxia or ARDS^c^) versus when it can be detrimental (eg, certain COPD exacerbations). Common misconception about fluid intake in post-operation patients: “Patients should remain NPO^d^ for as long as possible post-surgery.” Why it’s wrong*:* Prolonged NPO status can increase the risk of ileus, dehydration, and delays in recovery. Early feeding (as tolerated) is now recommended in many ERAS (enhanced recovery after surgery) protocols.
Medicine	Dengue immunity and waning antibody titres	I am a clinician educator in my university hospital. I am delivering a lecture to a group of medical students. I would like to convey the concept of waning immunity and the fact that having dengue once does not guarantee long-term immunity. Provide some common student misconceptions.	Explain serotype-specific immunity and the effect of waning antibody titres in dengue reinfection.	Common misconception on dengue immunity: “Having dengue once guarantees long-term immunity against it.” Why it’s wrong: Dengue is caused by four distinct serotypes (DENV1, DENV2, DENV3, and DENV4). Infection with one serotype only provides long-term immunity to that specific serotype. It does not confer complete protection against the others. Vaccines must balance immunity across all four serotypes. Common misconception on dengue immunity: “Once infected, dengue antibodies stay strong forever.” Why it’s wrong: Antibody levels wane over time, reducing neutralization and potentially allowing more severe disease upon reinfection.
Molecular Biology	DNA replication	I am teaching an introductory biology class to freshmen in Pharmacy. I am trying to convey the concept that DNA replication begins at multiple points of origin and DNA Polymerase uses dNTPs^e^ for DNA replication. Come up with likely misconceptions that students may have about these two concepts.	Explain that DNA replication begins at multiple points. Explain how dNTPs provide energy for phosphodiester bond formation during DNA synthesis.	Common misconception about DNA replication at multiple points (in eukaryotes): “Replication starts at only one point, like in bacteria.” Why it’s wrong: Students often generalize what they learn from prokaryotic replication (which starts at a single origin) to eukaryotic systems. Common misconception about dNTPs in DNA replication: “The dNTPs are added in their final form directly into DNA.” Why it’s wrong: Students may not understand that the energy for bond formation comes from the triphosphate tail of the dNTP, which is hydrolyzed during incorporation.
Microbiology	Viral spillover effects in humans, and viral evolution	I am an educator within my university’s microbiology and immunology department. I am delivering a lecture on SARS-CoV-2 spillover effects and viral evolution. Generate a list of plausible student misconceptions that I can debunk and explain.	Explain how evolutionary pressures might shape viral transmissibility without assuming linear directionality. Describe viral mutation as a process of random mutation.	Common misconception about SARS-CoV-2 spillover: “Viruses always evolve to become weaker over time.” Why it’s wrong: Evolution is not directional; some variants evolve for higher transmissibility, some for immune escape, and others may evolve increased or decreased virulence. Common misconception about viral evolution: “Viruses evolve intentionally to bypass immunity or treatments.” Why it’s wrong: Evolution is a process of random mutations and natural selection — there’s no ‘intent’ behind it.

a SpO2: oxygen saturation.

b COPD: chronic obstructive pulmonary disease.

c ARDS: acute respiratory distress syndrome.

d NPO: nil per os (nothing by mouth).

e dNTP: deoxynucleotide triphosphate.

Of interest are the inherent assumptions made by ChatGPT about a student’s prior knowledge in the subject. For example, in the molecular biology misconception on DNA replication (Table 1), ChatGPT assumes that students in an introductory molecular biology class can contrast DNA replication between prokaryotes and eukaryotes, despite the prompt only indicating misconceptions on multiple points of DNA replication. Such an assumption may not hold true in an average cohort. This may indicate a form of metacognitive hindsight bias intrinsic within GPT-related architectures. Educators could then include clarification prompts on the existing knowledge base of the target audience and modify AI-generated misconceptions to suit teaching needs.

In addition, educators can draw students’ attention to instances of hasty generalization within LLM-generated misconceptions such as in Table 1. For example, absolute qualifiers such as “all” (nursing prompt), “guarantees” (medical prompt), or “always” (microbiology prompt) imply universal causation with no exceptions. By explicitly highlighting these terms, educators can introduce counterexamples that challenge such overgeneralizations, providing a method for debunking misconceptions.

Finally, educators with prior experience of running past editions of the course in previous years could provide students’ examination answers as an input for LLM analysis, after anonymizing student answer scripts. This could clarify the scope and customize the misconceptions generated to a specific educator’s need. However, given excess specific input, the LLM runs a risk of factoring in irrelevant information, potentially leading to erroneous outputs. Educators could potentially mitigate this by limiting excessive irrelevant information and vetting the input contents to prevent overloading of the LLM’s context window.

## A 10-Step Guide for Implementing LLM-Augmented Misconception-Based CL

For educators who intend on implementing the framework in Figure 1 in practice, the following is a suggested 10-step guide with the concept of serotype-specific immunity in Table 1 used as an example.

### Educator-Led Steps

#### Step 1: Setup

Input lecture slides and/or reference textbook chapters into the LLM. Include specifics of your role (eg, clinician educator) and audience (eg, medical students) in the prompt.

In this step, educators should input lecture slides and/or reference textbook chapters into the LLM. Include specifics of your role (eg, clinician educator) and audience (eg, medical students) in the prompt.

#### Step 2: Review

Educators review the misconceptions provided. LLMs such as ChatGPT-4o may occasionally provide misconceptions based on content outside the scope of coverage.

#### Step 3: Tailor

Educators tailor the subsequent prompts to vary the length of misconceptions provided by the LLM. For example, if the concepts covered pertain to the nature of serotype-specific immunity and its relationship to long-term immunity such as antibody-dependent enhancement, a prompt instructing the LLM to integrate these concepts into a single misconception statement may be necessary. The integration of multiple related concepts into one misconception can test students’ attention to detail.

### Student-Derived Steps

#### Step 4: Setup

Upload anonymized student examination answers and lecture notes into an institutional pre-trained LLM chatbot.

#### Step 5: Role-play

Prompt the chatbot to: (1) assume the role of a junior medical student, (2) engage in a Socratic-style conversational dialogue, (3) ask questions individually and reply to responses with a follow-up question, and (4) provide some misconceptions within its train of thought based on the uploaded student examination answers.

Alternatively, if institutional chatbots are not available, ChatGPT-4o may be used. Only input lecture notes and/or reference textbook chapters and not student examination answers would be used in this case, as sending past student examination answers to current students may potentially infringe upon data privacy.

#### Step 6: Group

Depending on the cohort size and availability of educators, split the students into groups of 3‐5 to discuss the generated misconceptions for CL purposes. CL should preferably be implemented after students have gone through the content at least once, either through pre-reading or classroom instruction.

#### Step 7: Present

Student groups should conduct short presentations to identify and explain the LLM-generated misconceptions.

#### Step 8: Converse

Instruct the students to access the prompted institutional chatbot or ChatGPT-4o and initiate the conversation.

#### Step 9: Collaborate

Allow the students to work collaboratively in groups to address the questions posed by the LLM and “teach” it the concepts. Students could download the chat history or transcripts after the conclusion of the “lesson.”

#### Step 10: Assess

Students’ misconceptions could be assessed afterwards by reviewing their responses within the downloaded chat transcripts.

## Limitations and Directions for AI-Assisted Pedagogy in Health Care Education Studies 

It should be noted that only 2 quantitative studies [28,29] have been conducted on misconception-based reasoning and the wider misconception-based learning literature within the context of health care education. More than half of these studies are focused on the fields of Newtonian physics, genetically modified organisms, and evolution [30]. While misconception-based learning was determined to be robust across disciplines independent of specific text design [31], generalizing misconception-based learning’s efficacy from other fields to health care education may not be fully applicable. More studies are required to examine the impact of this pedagogical method on student outcomes in health care education.

A common methodological critique of this pedagogical approach is its vulnerability to pre-test priming effects. In typical designs, students complete a pre-test before exposure to the instructional intervention and a post-test afterward. However, evidence suggests that notifying students about an impending test may activate prior knowledge and trigger conceptual change, making it challenging to distinguish between the effects of pre-test priming and those of LLM-assisted, misconception-based CL. To address this, researchers could track individual student responses and apply statistical controls—such as paired *t* tests or ANCOVA analyses—to adjust for pretest influences and better isolate the intervention’s true impact.

CL applied in LLM-generated misconception generation assumes that there is a form of homogeneity in conceptual understanding within and between groups. In a group comprised only of weaker students, false explanations of the LLM-generated misconceptions may be negatively reinforced instead. While ensuring the distribution of students with varying backgrounds and degrees of conceptual understanding within groups may be ideal to fully capitalize on the benefits of CL, such methods are difficult to implement in practice and are a key limitation of this pedagogical method.

Given the limited number of studies present to examine AI-assisted pedagogy in health care education, multiple avenues for follow-up research studies exist. Few studies have assessed cognitive attitudes and student perspectives on CL using AI [32]. Importantly, the critical nature of such studies stems from the increasingly interconnected working environment. Studying cognitive attitudes on CL using AI from students’ perspectives could better associate AI technology implementation in the classroom with student outcomes.

Additionally, there has been a lack of medium- to long-term research on student outcomes using AI [33]. The prevalence of AI technologies and its impact on the educational setting have far-reaching implications for changing pedagogy and student learning. However, since the rise of LLMs to prominence in 2023, studies have largely been cross-sectional, capturing only a snapshot of AI’s influence at a specific point in its rapid development.

Cross-sectional studies are likely only able to capture a snapshot of AI’s impact on student learning at a specific point of time in AI’s development. Such studies often overlook how a student’s learning trajectory evolves alongside advances in AI itself. For example, an educational experience supported by GPT-3 would likely differ substantially from one shaped by GPT-4o, which introduced capabilities such as image generation. Longitudinal, mixed-methods studies are needed to track these evolving dynamics, providing deeper insights into both shifts in student perceptions and longer-term learning outcomes as AI technologies continue to evolve. Such an approach could also assist to identify key factors that support or hinder the long-term implementation of AI in teaching practice.

Yet, it should be noted that the fast-evolving nature of LLMs—which at the time of writing include Gemini 2.5 and the upcoming GPT-5 by OpenAI—could impact the reproducibility of generated misconceptions. With larger token sizes and greater architecture complexity, prompts provided to newer models may not produce the same outputs. Future educational research involving LLMs could ensure the reporting of the specific model version used, sampling conditions, and prompts used for an effort towards greater reproducibility. While the stochastic nature of LLMs carries a risk of low reproducibility of outputs with the same prompt, the role of educators to mitigate such a risk could be to verify and vet the LLM-generated outputs. Thus, LLM-generated misconceptions should serve to complement curriculum design, and the educator should not be removed from the process.

Additionally, greater data privacy risk comes with the evolving dynamics of new LLM models. The extent to which newer models retain data inputs by users through larger context windows are not well understood. Therefore, such risks could be mitigated by educators through anonymization and the removal of original file metadata. On the end of educational technology developers, methods to protect data security such as adversarial attack testing or encryption specific to LLM modalities could be explored [34].

Finally, explicit indications of the misconception and subsequent explanations by educators may not always work if the misconception is entrenched in the students’ mental models. Greater emphasis needs to be placed on understanding the root of the falsehood, perhaps through analysis of past lived experiences of students that led to this network of beliefs, to facilitate effective conceptual change. Addressing misconceptions in education would therefore likely not be a one-size-fits-all solution. Supplementing refutation-based learning with other educational approaches such as concept maps and audiovisual representation may improve coherence of concept representation [29].

## Conclusion – LLM’s Potential for Augmenting Misconception-Based Learning in Health Care Education

Misconceptions, which could stem from naive misinformation, have implications on critical thinking and student learning. Yet, when leveraged intentionally—for example, through LLM-augmented, misconception-based CL—they can catalyze transformative shifts in teaching practice. This approach not only deepens students’ understanding of subject matter but also provides a valuable teaching opportunity for exploring both the benefits and limitations of LLMs.

Additionally, the framework serves as a springboard for future empirical research, generating several potentially testable hypotheses. For example, does pre-bunking with LLM-generated misconceptions elicit stronger propensity for conceptual accommodation as compared to educator-generated refutation text? Does CL around LLM-generated misconceptions improve reasoning accuracy and quality compared to individual learning? Does LLM-generated misconceptions change classroom dynamics for CL? These are important questions for identifying and testing the value of LLMs in misconception-based CL.

However, the true value of LLMs in misconception-based learning exists not to replace educator involvement and expertise. Rather, its purpose is to augment student learning with personalized feedback, synthesize “edge-case” concepts across topics, and reduce the time taken for educators by supplementing misconceptions. The educator’s role is still important in context-setting, the facilitation of CL, and critical review of the AI output. Ultimately, the proposed framework positions LLMs not as an endpoint for teaching and learning, but to foster self-directed, critical, and reflective thinking.

## Abbreviations

AI: artificial intelligence
CL: collaborative learning
LLM: large language model
MBC: misconception-based collaborative
